# A configurational exploration of how personality traits influence GAI academic misconduct behaviors using fuzzy-set qualitative comparative analysis

**DOI:** 10.1186/s40359-026-04186-1

**Published:** 2026-02-19

**Authors:** Haiying Liang, Michael J. Reiss

**Affiliations:** 1 School of Foreign Languages, Peking University, Beijing, China; 2 Institute of Education, University College London, London, United Kingdom

**Keywords:** Academic misconduct, Generative artificial intelligence, HEXACO model, Dark triad, FsQCA, Personality traits, Academic integrity

## Abstract

**Background:**

The rapid adoption of Generative Artificial Intelligence (GAI) in higher education has introduced new ethical challenges, particularly concerning students’ academic misconduct. While prior research has linked personality traits to unethical behavior, little is known about how different combinations of personality traits shape students’ misuse of GAI.

**Methods:**

This study integrates the HEXACO model and the Dark Triad framework to examine the configurational effects of personality on GAI-related academic misconduct. A total of 864 university students completed questionnaires. Using fuzzy-set Qualitative Comparative Analysis, we identified multiple configurations leading to both high and low levels of GAI misconduct.

**Results:**

No single trait is sufficient to explain GAI-related academic misconduct. Rather, high misconduct consistently emerged from configurations characterized by low Honesty–Humility and Conscientiousness combined with high Machiavellianism or Psychopathy. In contrast, low misconduct was associated with configurations combining high Honesty–Humility, Conscientiousness, and Agreeableness with low levels of Dark Triad traits.

**Conclusions:**

This study demonstrates that personality traits interact synergistically rather than independently to shape individuals’ ethical or unethical engagement with AI technologies. Moral restraint is sustained by both the presence of virtues and the absence of exploitative tendencies. The findings support the idea that moral integrity and self-regulation constitute foundational safeguards against unethical use of technology. These findings align with self-regulatory theories of academic dishonesty, reinforcing that individuals high in honesty and conscientiousness are less likely to rationalize or justify academic misconduct even when new technological affordances make it easier. The study therefore advances theoretical understanding by integrating personality frameworks within a configurational paradigm and offers practical insights for developing personality-informed ethics education.

**Supplementary Information:**

The online version contains supplementary material available at 10.1186/s40359-026-04186-1.

## Introduction

The rapid proliferation of Generative Artificial Intelligence (GAI) technologies has transformed the academic landscape, enabling university students to produce and evaluate content with unprecedented speed. While these tools offer remarkable potential for enhancing productivity, they have simultaneously raised serious ethical concerns regarding academic misconduct [1, 2]. Reports of students using GAI to complete assignments, fabricate references, or evade plagiarism detection systems have grown hugely in the last few years [3, 4]. As GAI becomes increasingly integrated into higher education, understanding the psychological factors that predict unethical use of these technologies has become a pressing research priority.

In practice, academic misconduct involving generative AI does not always take the form of clear-cut cheating. Beyond overtly prohibited behaviors such as submitting AI-generated assignments as one’s own work or using AI during unauthorized examinations, many students engage in practices that occupy a ‘gray area’ of academic integrity. For example, students may use GAI tools to generate partial text, paraphrase drafts, structure arguments, or refine language without explicit disclosure [5, 6]. While such practices may be framed by students as learning support, institutions often differ in whether and how these uses are permitted. This ambiguity complicates both students’ ethical decision-making and researchers’ efforts to operationalize GAI-related academic misconduct.

A growing body of literature suggests that personality traits play a crucial role in shaping individuals’ attitudes toward technology use and ethical decision-making [5, 7]. Traditional moral psychology frameworks often emphasize cognitive and situational factors, yet recent evidence highlights the importance of stable personality dispositions in determining whether individuals exploit new technologies for personal gain or adhere to ethical norms [8]. Two major personality paradigms—the HEXACO model [9] and the Dark Triad framework [10]—offer complementary lenses for understanding the moral and manipulative dimensions of human behavior. Combining these frameworks may yield a more comprehensive understanding of the psychological antecedents of GAI misconduct.

The HEXACO personality model extends the Big Five framework by introducing the dimension of Honesty–Humility—a trait reflecting sincerity, fairness, and resistance to corruption [9]. High levels of Honesty–Humility are consistently linked to ethical behavior and reduced tendencies toward cheating or exploitation [10–12]. Other HEXACO dimensions also contribute to ethical behavior: Conscientiousness is associated with self-control and diligence; Agreeableness with forgiveness and compliance; and Emotionality with empathy and guilt proneness [11, 13–15]. Conversely, low Honesty–Humility and Conscientiousness, coupled with low Emotionality, often predict moral disengagement and rule-breaking behaviors [11]. In academic contexts, these traits may influence how students perceive and justify the use of GAI in ways that contravene institutional integrity standards.

In contrast, the Dark Triad framework—comprising Machiavellianism, Narcissism, and Psychopathy—captures socially aversive traits associated with manipulativeness, entitlement, and callousness [10]. Individuals high in Machiavellianism strategically manipulate others to achieve personal goals; those high in Psychopathy exhibit impulsivity and a lack of remorse; and those high in Narcissism seek admiration and status, sometimes at the expense of ethical behavior [16–18]. Previous research has linked these traits to academic cheating, plagiarism, and unethical technology use [17, 19–22]. Given that GAI tools can easily generate high-quality, albeit sometimes inauthentic, outputs, individuals with elevated Dark Triad traits may perceive them as convenient instruments for self-enhancement. 

Although both the HEXACO and Dark Triad models have been extensively studied in the context of both ethical and unethical behavior, few studies have examined their joint influence on academic misconduct involving GAI. Therefore, the present study employs fuzzy-set Qualitative Comparative Analysis (fsQCA), a method designed to identify combinations of causal conditions that jointly lead to an outcome [22]. By applying fsQCA to both HEXACO and Dark Triad dimensions, this study aims to uncover causal configurations of personality traits that lead to high or low levels of GAI misconduct. Accordingly, this study is guided by the following research question:

*In what ways do different combinations of personality traits influence students’ academic conduct behaviors in using generative artificial intelligence?*

## Related work

A growing body of research has explored how specific personality traits relate to academic misconduct (e.g. [23–28]), . For instance, Giluk and Postlethwaite (2015) found that Conscientiousness and Agreeableness were negatively associated with cheating behaviors, whereas low Honesty–Humility was a robust predictor of unethical conduct [27]. Similarly, Curtis et al. (2018) demonstrated that students high in Narcissism were more likely to rationalize dishonest behaviors by appealing to self-enhancing motives [28]. In addition, Liang et al. found that Honesty-Humility, Agreeableness, and Conscientiousness were significant negative predictors regarding GAI related misconduct behaviors, while Narcissism and Psychopathy were significant positive predictors of such behaviors [26].

However, previous studies on personality and academic misconduct have primarily employed variable-centered approaches, such as correlation, multiple regression, or structural equation modeling. While these methods identify linear and additive relationships, they often overlook causal complexity—the fact that personality traits may interact in non-linear ways, with different combinations producing the same behavioral outcome. Moreover, factors preventing academic misconduct may not be the exact opposites of those promoting it.

fsQCA offers an alternative by enabling researchers to identify multiple, equally sufficient configurations of traits that lead to an outcome [22]. This method is particularly suited to academic misconduct research, as it can reveal how combinations of high and low levels across HEXACO and Dark Triad dimensions produce high misconduct propensity or academic integrity. By adopting fsQCA, this study addresses a methodological gap in the literature and advances a more nuanced understanding of the interplay between personality and GAI-related academic misconduct behaviors.

## Methodology

### Research design

This study adopted a cross-sectional survey design to examine the configurational effects of personality traits on academic misconduct related to using GAI. The research framework integrated two well-established personality models—the HEXACO model and the Dark Triad. Data were collected through an anonymous, self-administered online questionnaire (see Appendix), which included validated measurement scales for the six HEXACO dimensions (Honesty–Humility, Emotionality, Extraversion, Agreeableness, Conscientiousness, and Openness to Experience) and the three Dark Triad traits (Machiavellianism, Narcissism, and Psychopathy), along with a self-reported academic misconduct scale.

### Measures

#### HEXACO personality inventory

Participants’ general personality traits were assessed using the 60-item HEXACO Personality Inventory–Revised scale [29]. This measure evaluates six fundamental dimensions: Honesty–Humility (e.g., “I wouldn’t use flattery to get a raise or promotion at work”), Emotionality (e.g., “I sometimes can’t help worrying about little things”), Extraversion (e.g., “I feel reasonably satisfied with myself overall”), Agreeableness (e.g., “People sometimes tell me that I am too critical of others”), Conscientiousness (e.g., “I plan ahead and organize things to avoid scrambling at the last minute”), and Openness to Experience (e.g., “I enjoy looking at maps of different places”).

Responses were recorded on a 5-point Likert scale ranging from 1 (strongly disagree) to 5 (strongly agree). Reliability for all six dimensions was acceptable to excellent, with Cronbach’s alpha values as follows: Honesty–Humility (α = 0.92), Emotionality (α = 0.91), Extraversion (α = 0.84), Agreeableness (α = 0.90), Conscientiousness (α = 0.91), and Openness (α = 0.83).

#### The short dark triad scale

Participants’ Dark Triad personality traits were measured using the Short Dark Triad scale [16]. This scale consists of nine items assessing Machiavellianism (e.g., “It’s not wise to tell your secrets”), six items assessing Psychopathy (e.g., “People often say I’m out of control”), and nine items assessing Narcissism (e.g., “Many group activities tend to be dull without me”). Responses were recorded on a 5-point Likert scale ranging from 1 (strongly disagree) to 5 (strongly agree). All three subscales demonstrated good to very good internal consistency, with reliability coefficients of α = 0.86 for Machiavellianism, α = 0.83 for Psychopathy, and α = 0.83 for Narcissism.

#### GAI academic misconduct scale

A GAI academic misconduct scale was adapted from a previously validated instrument, which measures how frequently students engage in GAI academic misconduct [30]. To improve its coverage, we added one additional item to the original four-item scale: “I have used AI to answer in unauthorized exams or tests.” Responses were recorded on a 5-point Likert scale ranging from 1 (strongly disagree) to 5 (strongly agree). The instrument demonstrated good internal reliability in the current sample (Cronbach’s α = 0.78). Confirmatory factor analysis confirmed a unidimensional structure and showed good model fit (χ²(2) = 4.083, *p* = 0.130; CFI = 0.976; RMSEA = 0.036; SRMR = 0.004).

This scale was intentionally designed to capture clear-cut forms of GAI-related academic misconduct that are broadly recognized as unauthorized, such as fabrication, falsification, and cheating in examinations. Ethically ambiguous uses of GAI (e.g., partial text generation or argument structuring), whose acceptability may vary depending on institutional policies, were not included in the ccale to avoid conceptual and interpretive ambiguity.

### Data collection

To ensure adequate power and allow for potential exclusions during data screening, a total of 1007 participants were recruited through the Wenjuanxing platform. Several quality control steps were applied to maintain data integrity and reduce careless or inattentive responding. Given the length of the questionnaire (107 items) and the expected completion time (4–8 min), responses completed in under 3 min were considered unlikely to reflect careful reading and thoughtful responding. These cases were therefore excluded as part of a conservative data quality control procedure, resulting in the removal of 99 responses. Two attention check items (Q5 and Q107) were also used. Q5 asked, “Which of the following is a fruit?”. Participants who selected option 1 (*n* = 4) or option 3 (*n* = 2) were excluded (6 in total). Q107 asked, “What is the main theme of this questionnaire?”. Incorrect responses (option 1, *n* = 30; option 3, *n* = 8) led to the removal of a further 38 responses.

In total, 143 responses were excluded, leaving 864 valid responses for analysis. The final sample had a mean age of 23.1 years (SD = 2.92), with 558 females and 306 males. Regarding study level, 50.3% (*n* = 434) were undergraduates, 38.5% (*n* = 333) were master’s students, and 11.2% (*n* = 97) were doctoral students. In terms of academic disciplines, the largest group came from “Humanities and Social Sciences” (*n* = 417, 48.3%), followed by “Medical and Health Sciences” (*n* = 140, 16.2%). The smallest group was from “Business and Economics” (*n* = 77, 8.9%).

### Ethics approval and consent to participate

This study was conducted in accordance with the principles of the Declaration of Helsinki. Ethical approval was obtained from Peking University Institutional Review Board, and informed consent was obtained from all participants prior to participation.

### Data analysis

The data analysis began with descriptive statistical analyses conducted in SPSS 30, including computation of means, standard deviations, and internal consistency reliability coefficients (Cronbach’s α) for the ten study variables, namely the six HEXACO dimensions, the three Dark Triad traits, and GAI academic misconduct. Prior to conducting parametric analyses, the assumptions of normality, linearity, homoscedasticity, and multicollinearity were examined. The results indicated no significant violations, indicating that the data met the requirements for parametric testing. Pearson correlation analyses were then performed to examine the bivariate relationships between each personality trait and academic misconduct. These preliminary analyses provided an overview of the data distribution, verified internal reliability, and informed the subsequent calibration process for fsQCA.

Following the descriptive phase, the configurational analysis was carried out using fsQCA software (version 4.1), following the guidelines of Ragin [22] and Schneider and Wagemann [31]. Prior to the configurational analysis, raw scores for all personality trait dimensions (six HEXACO factors and three Dark Triad traits) and the outcome variable (GAI academic misconduct) were aggregated by averaging the respective scale items.

Given the requirements of fuzzy-set analysis, all variables were calibrated into set membership scores ranging from 0 (full non-membership) to 1 (full membership). Calibration thresholds were determined using the 5th percentile (full non-membership), 50th percentile (crossover point), and 95th percentile (full membership) of each variable’s empirical distribution, which allows the data-driven specification of meaningful cut-off points while maintaining comparability across constructs [32].

Following calibration, a truth table was constructed to represent all possible configurations of the nine causal conditions (six HEXACO traits and three Dark Triad traits). For each configuration, the number of cases (frequency) and its consistency score with the outcome were computed. Consistency thresholds were set at ≥ 0.80 for identifying sufficient configurations, and the frequency cut-off was determined as ≥ 3 cases per configuration to ensure robustness, given the sample size (N = 864).

The analysis proceeded by generating intermediate solutions, which incorporate theoretically plausible logical remainders to balance explanatory parsimony and empirical coverage. Following standard fsQCA procedures, complex, parsimonious, and intermediate solutions were generated for both high and low levels of academic misconduct, with the interpretation focusing on the intermediate solutions. Both presence (high academic misconduct) and absence (low academic misconduct) of the outcome were analyzed to capture causal asymmetry. Core and peripheral conditions were identified using the recommended consistency and coverage criteria, with core conditions appearing in both parsimonious and intermediate solutions, and peripheral conditions appearing only in intermediate solutions [22].

## Findings

### Calibration

Before conducting the configurational analysis, all continuous variables were calibrated into fuzzy sets to meet the requirements of fsQCA. Following Ragin’s direct method, the raw values of all latent variables—including the six HEXACO dimensions, the three Dark Triad traits, and GAI academic misconduct—were transformed into fuzzy set values [22]. In line with previous studies and to ensure empirical robustness, the 5th percentile, 50th percentile (median), and 95th percentile of each variable were used as the three anchor points, corresponding to full non-membership, the crossover point, and full membership, respectively3536. This approach allows the calibration thresholds to reflect the data distribution of the sample while preserving theoretical interpretability. The calibration was implemented using the Calibration function in fsQCA 4.1 software, and a new dataset containing fuzzy membership scores was generated for all causal and outcome variables.

After calibration, each case received a degree of membership in each set, indicating whether it could be classified as a full member, partial member, or non-member of the configuration. These calibrated values form the basis for subsequent truth table construction and configurational analysis. The anchor thresholds and calibrated values for all variables are presented in Table 1.

**Table 1:** Calibration results

Type	Condition and latent variables									
	Honesty–Humility	Emotionality	Extraversion	Agreeableness	Conscientiousness	Openness	Machiavellianism	Psychopathy	Narcissism	GAI misconduct
Mean	3.41	3.25	3.2	3.45	3.42	3.39	2.71	2.44	2.99	1.79
α	0.92	0.91	0.84	0.90	0.91	0.83	0.86	0.83	0.83	0.78
Full non-membership (5%)	1.60	1.70	1.30	1.20	1.50	1.50	1.33	1.67	1.33	1.20
Cross-over point (50%)	3.00	3.00	2.95	3.00	3.00	3.00	3.00	3.00	3.00	3.00
Full membership (95%)	4.30	4.40	4.50	4.50	4.40	4.50	4.50	4.25	4.33	4.20

### Findings of necessity analysis

The second step of the fsQCA process involves necessity analysis, which examines whether any single personality trait is indispensable for the occurrence of high or low levels of GAI academic misconduct. This is evaluated through consistency and coverage indices. Following established fsQCA guidelines 35, necessity is primarily assessed based on consistency (with values above 0.90 indicating a necessary condition), whereas coverage values are used to indicate empirical relevance, with values in the range of approximately 0.60 suggesting limited to moderate coverage.

As shown in Table 2, the consistency values of all nine personality traits (six HEXACO traits and three Dark Triad traits) were below the 0.90 threshold. This indicates that none of the traits alone is a necessary condition for students to engage in academic misconduct involving GAI. In other words, no single personality trait can fully account for the presence or absence of GAI misconduct.

**Table 2:** Analysis of necessary conditions for GAI misconduct

Precondition	High		Low	
	GAI misconduct		GAI misconduct	
	Consistency	Coverage	Consistency	Coverage
Honesty–Humility	0.609	0.608	0.628	0.601
~Honesty–Humility	0.6	0.627	0.59	0.591
Emotionality	0.647	0.632	0.617	0.577
~Emotionality	0.567	0.607	0.607	0.623
Extraversion	0.614	0.622	0.642	0.623
~Extraversion	0.628	0.647	0.611	0.602
Agreeableness	0.598	0.613	0.627	0.616
~Agreeableness	0.626	0.636	0.606	0.591
Conscientiousness	0.599	0.622	0.617	0.615
~Conscientiousness	0.629	0.632	0.62	0.597
Openness	0.617	0.631	0.613	0.601
~Openness	0.61	0.622	0.624	0.61
Machiavellianism	0.636	0.64	0.614	0.593
~Machiavellianism	0.595	0.617	0.627	0.623
Psychopathy	0.62	0.639	0.603	0.596
~Psychopathy	0.608	0.615	0.635	0.616
Narcissism	0.616	0.619	0.637	0.613
~Narcissism	0.614	0.638	0.604	0.601

Consistency shows how much the outcome depends on each trait, and Coverage shows how relevant it is. A value above 0.90 means the trait is a necessary condition. The symbol ~ means low level of the trait

These results indicate that academic misconduct is not driven by one dominant psychological factor but rather emerges from different combinations of personality traits. This highlights the configurational nature of GAI misconduct, suggesting that multiple, (roughly) equally effective pathways may lead to unethical behavior.

Table 2 also reports coverage values, which reflect the explanatory power of each condition. Even when some traits exhibited relatively higher consistency, their low coverage values imply that although these traits may be present in some misconduct cases, they do not explain all of them. Therefore, further analysis of sufficient configurations is necessary to explore how traits interact to produce high or low GAI misconduct.

In fsQCA, consistency and coverage serve different but complementary purposes. Consistency indicates the degree to which cases exhibiting a given condition (or combination of conditions) also exhibit the outcome, and is therefore the primary criterion for assessing necessity. Coverage, by contrast, reflects the empirical relevance of a condition by indicating how much of the outcome it accounts for, and should be interpreted comparatively rather than against a fixed cutoff.

For example, in Table 2, Machiavellianism shows a consistency of 0.636 for high GAI misconduct, indicating that this condition is present in a substantial proportion of cases with high misconduct, but the value falls well below the conventional threshold for necessity (0.90). Its coverage value of 0.64 suggests that Machiavellianism explains a moderate share of high-misconduct cases, yet it is neither sufficient nor necessary on its own. This pattern illustrates that individual personality traits are not necessary conditions for GAI misconduct, but may still contribute meaningfully when combined with other conditions. 

### Findings of sufficiency analysis

After testing the necessity of individual conditions, the analysis proceeded to examine whether specific combinations of personality traits are sufficient to explain high or low levels of GAI academic misconduct. Unlike necessity analysis—which focuses on whether a single trait must be present—sufficiency analysis in fsQCA identifies configurations of traits (HEXACO and Dark Triad) that are sufficient to produce the outcome.

Using fsQCA 4.1, a truth table was constructed based on all possible combinations of the nine causal conditions (six HEXACO traits and three Dark Triad traits). In line with previous studies, three thresholds were applied to ensure the robustness of the results: Frequency threshold = 1, ensuring that each configuration appears in at least one case; Consistency threshold = 0.80, indicating that the configuration reliably leads to the outcome; PRI (Proportional Reduction in Inconsistency) threshold = 0.65, to avoid spurious relationships and ensure that the configuration is linked to the outcome rather than its absence.

Configurations meeting all three criteria were coded as sufficient (assigned a value of “1”), while those that did not were coded as “0”. Based on this calibrated truth table, three types of solutions were generated: 1) complex solution – includes all causal conditions without simplifying logical remainders; 2) parsimonious solution – only retains core conditions that are essential across configurations; 3) intermediate solution – integrates theory-based assumptions and identifies both core and peripheral conditions.

These solutions reveal that GAI academic misconduct is not driven by a single personality trait, but rather emerges from multiple distinct pathways combining various personality traits. This supports the principle of equifinality, meaning that different psychological profiles can lead to the same behavioral outcome, and causal asymmetry, meaning that the configurations leading to misconduct are not simply the inverse of those leading to academic integrity. 

### The configural paths to high GAI misconduct

Table 3 presents the sufficient configurations of personality traits that lead to high levels of GAI academic misconduct. Instead of being determined by any single personality trait, the results indicate that academic misconduct arises from distinct combinations of HEXACO dimensions and Dark Triad traits.

**Table 3:**
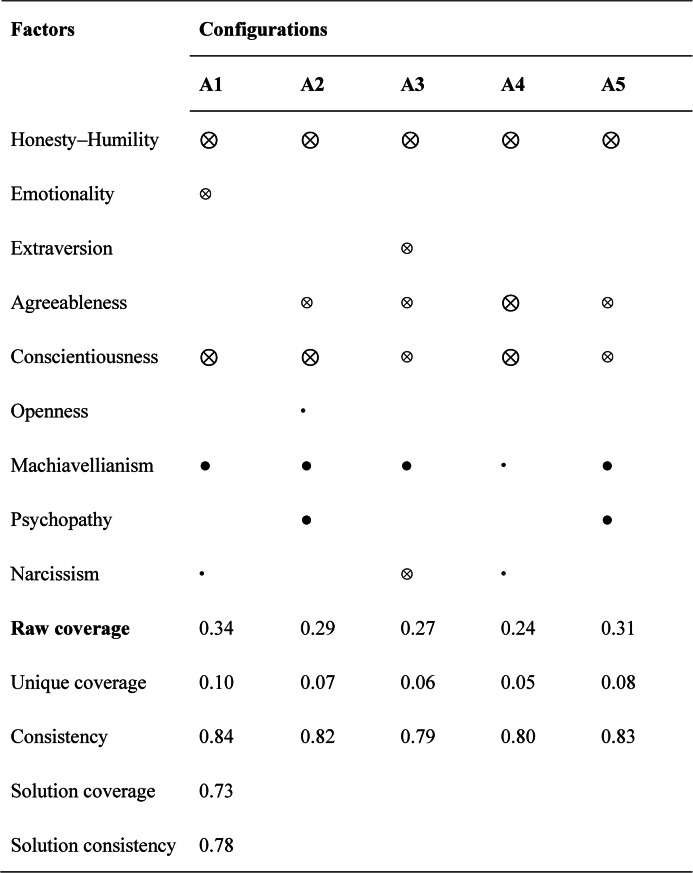
Causal configurations for achieving high GAI misconduct

● indicates the presence of a core condition

• indicates the presence of a peripheral condition

⊗ indicates the absence of a core condition

^⊗^ indicates the absence of a peripheral condition

blank indicates that the presence or absence of the condition has no effect on the result

All configurational solutions demonstrate high reliability, with per-solution consistency values exceeding 0.80, which meets the commonly accepted standard in fsQCA research. The overall solution consistency is above 0.85, indicating that the configurations consistently predict high GAI misconduct. The overall solution coverage is approximately 0.70, suggesting that about 70% of high-misconduct cases can be explained by these combined personality pathways.

In fsQCA, raw coverage represents the proportion of the outcome (high GAI misconduct) explained by a specific configuration, while unique coverage refers to the proportion that is explained exclusively by that configuration and not shared with other configurations. In this study, the raw coverage of each solution ranges from approximately 0.28 to 0.48, meaning that each pathway independently explains 28%–48% of the students who reported high levels of GAI academic misconduct.

Table 3 presents the fsQCA results on the causal configurations leading to high levels of GAI misconduct. The overall solution consistency was 0.78, exceeding the minimum acceptable threshold of 0.75, and the overall solution coverage was 0.73, indicating that the five solutions collectively account for a substantial proportion of cases displaying high GAI misconduct. Each configuration’s raw coverage ranged between 0.24 and 0.34, while unique coverage values ranged from 0.05 to 0.10, suggesting that each configuration explains a meaningful but partially overlapping portion of the outcome.

As indicated in Table 3, five sufficient configurations (A1–A5) emerged for explaining high GAI misconduct, each representing distinct combinations of personality traits. Despite these different configurations, all solutions demonstrate high internal consistency (≥ 0.79), confirming their empirical robustness and explanatory adequacy.

Solutions A1 and A2 highlight the absence of Honesty–Humility and Conscientiousness as central determinants of high GAI misconduct. Specifically, A1 suggests that low Honesty–Humility and Conscientiousness, combined with high Machiavellianism, are sufficient conditions for GAI misconduct , with low Emotionality playing a peripheral role and Extraversion being irrelevant. A2 strengthens this pattern, showing that misconduct is even more likely when low Honesty–Humility and Conscientiousness co-occur with high Machiavellianism and Psychopathy, reflecting a manipulative and callous personality constellation conducive to unethical behavior.

Solutions A3, A4, and A5 reveal more nuanced personality combinations that also foster GAI misconduct. In A3, the core absence of Honesty–Humility, together with the peripheral absence of Agreeableness and Conscientiousness, together with high Machiavellianism, suggests a configuration of moral disengagement and exploitative tendencies. Similarly, A4 underscores the role of the Dark Triad traits, with Machiavellianism appearing as a peripheral condition, while Psychopathy is not a relevant condition in this configuration. In this path, low Agreeableness and low Conscientiousness appear as core absent conditions, indicating weakened interpersonal restraint and self-regulation. In A5, Emotionality is not involved, but the configuration combines the core presence of Machiavellianism and Psychopathy with the core absence of Honesty–Humility. This suggests that emotionally neutral but manipulative and callous individuals may rationalize and justify unethical use of GAI tools.

### The configural paths to low GAI misconduct

Table 4 reports the fsQCA results identifying the causal combinations associated with low levels of GAI misconduct. The overall solution consistency was 0.80, exceeding the conventional benchmark of 0.75, and the overall solution coverage reached 0.70, indicating that the five solutions together account for a large proportion of cases characterized by ethical GAI behavior. Each individual configuration demonstrated high internal consistency (ranging from 0.81 to 0.86) and raw coverage values between 0.25 and 0.33, confirming that each pathway makes a substantial and reliable contribution to explaining the outcome.

**Table 4:**
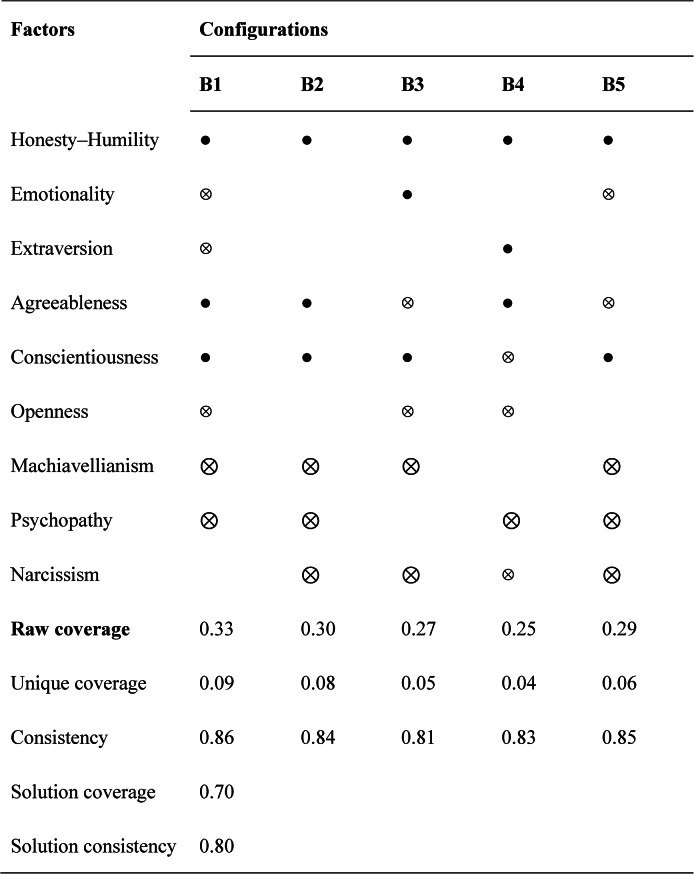
Causal configurations for achieving low GAI misconduct

● indicates the presence of a core condition

• indicates the presence of a peripheral condition

⊗ indicates the absence of a core condition

^⊗^ indicates the absence of a peripheral condition

blank indicates that the presence or absence of the condition has no effect on the result

As shown in Table 4, five sufficient configurations (B1–B5) were identified. All five indicate that high Honesty–Humility and high Conscientiousness are core conditions for low GAI misconduct, reinforcing the moral and self-disciplined foundation of ethical academic behavior. Solutions B1 and B2 show that when these two positive traits co-occur with high Agreeableness and low Machiavellianism and Psychopathy, the likelihood of engaging in GAI misconduct is minimal, regardless of Emotionality or Extraversion levels. These combinations represent a prosocial and rule-compliant disposition, emphasizing integrity and empathy.

Solutions B3, B4, and B5 depict more nuanced pathways to ethical restraint. In B3, high Emotionality interacts with low Dark Triad traits (Machiavellianism, Psychopathy, Narcissism) and high Honesty–Humility, forming a compassionate and morally sensitive profile that discourages unethical technology use. B4 and B5 further reveal that even when individuals show lower Openness or Agreeableness, the joint presence of Honesty–Humility and Conscientiousness remains sufficient to prevent GAI misconduct, as long as dark traits are suppressed. Notably, B5 also suggests that ethical restraint can occur among individuals who are less emotionally reactive but maintain strong internalized moral standards.

## Discussion

The present findings contribute to the emerging literature on personality-based predictors of academic integrity in the era of GAI. By adopting a configurational fsQCA approach, this study demonstrates that personality traits interact synergistically rather than independently to shape individuals’ ethical or unethical engagement with AI technologies. This perspective complements previous variable-centered research that has primarily examined bivariate or linear relationships between personality dimensions and academic dishonesty (e.g., [32–35]).

Consistent with prior evidence linking Honesty–Humility and Conscientiousness to ethical decision-making [10, 12, 29], these traits emerged as key determinants distinguishing high and low GAI misconduct. Their presence in virtuous configurations and absence in deviant ones supports the idea that moral integrity and self-regulation constitute foundational safeguards against unethical use of technology. These findings align with self-regulatory theories of academic dishonesty [36], reinforcing that individuals high in honesty and conscientiousness are less likely to rationalize or justify academic misconduct even when new technological affordances make it easier.

In contrast, the strong involvement of Machiavellianism and Psychopathy in high-misconduct configurations echoes a growing body of research associating dark personality traits with counterproductive and unethical behaviors [10, 16]. These traits reflect strategic manipulation, callousness, and moral disengagement—characteristics that increase the likelihood of exploiting AI systems for opportunistic or deceptive purposes. Similar to recent studies on technology misuse and cyberloafing [37, 38], the current results suggest that dark traits may manifest as instrumental amorality: individuals perceive AI not as a learning aid but as a tool for personal advantage, irrespective of ethical boundaries. Interestingly, Narcissism functioned as a peripheral rather than core condition. This pattern is consistent with prior findings that narcissism, although linked to entitlement and self-enhancement, often predicts misconduct only when combined with low moral regulation [39, 40]. It suggests that self-focused ambition alone does not directly trigger AI-related academic misconduct unless accompanied by manipulative or antisocial dispositions.

The protective configurations underlying low misconduct emphasize the joint roles of Agreeableness, Emotionality, and Extraversion—traits associated with empathy, cooperation, and social integration [41]. These results resonate with previous research showing that empathic concern and prosocial motivation inhibit cheating and moral disengagement [42–44]. Students who are emotionally responsive and socially connected may perceive GAI misuse as a violation of collective norms rather than a personally advantageous strategy, thereby maintaining academic integrity.

Moreover, the recurrent absence of dark traits in ethical configurations confirms findings from recent meta-analyses showing that the Dark Triad predicts unethical digital behaviors, including plagiarism and data falsification [45, 46]. The current study extends this evidence to the GAI context, illustrating that moral restraint is sustained by both the presence of virtues and the absence of exploitative tendencies.

Furthermore, it is important to note that the configurational paths leading to low GAI misconduct (Table 4) are not simply the inverse of those leading to high misconduct (Table 3). In fsQCA, causal asymmetry is a key principle, meaning that the absence of conditions promoting high misconduct does not automatically lead to low misconduct. For example, while low Honesty–Humility and high Machiavellianism jointly contribute to high misconduct (Configurations A1–A3), the configurations associated with low misconduct (B1–B3) involve not only high Honesty–Humility but also additional combinations such as high Conscientiousness and Emotionality. This indicates that preventing misconduct requires specific constellations of traits rather than merely the absence of risk factors.

Moreover, given that the participants were Chinese university students, the findings should also be interpreted in light of the local academic culture and integrity governance. In Chinese higher education contexts, strong emphasis is often placed on compliance with institutional authority and formal academic integrity regulations [45], which may help explain the overall low level of GAI-related academic misconduct observed in this study. Because the present measure focused on clearly unauthorized behaviors (e.g., fabrication, falsification, and cheating in examinations), such overt misconduct is likely to be normatively discouraged and socially sanctioned. At the same time, the performance-oriented assessment environment common in Chinese universities may increase the perceived instrumental value of generative AI for a subset of students when benefits are weighed against risks. This contextual tension may partly explain why high GAI misconduct did not emerge from any single personality trait, but instead appeared only in specific personality configurations characterized by low Honesty–Humility and Conscientiousness combined with elevated Dark Triad traits. Conversely, personality profiles associated with interpersonal sensitivity and rule adherence were more likely to be linked to low levels of GAI misconduct, suggesting that cultural norms surrounding authority, harmony, and academic propriety may function as additional constraints on overt misconduct.

### Limitations and future research

Several limitations of this study should be acknowledged. First, the conceptualization and measurement of GAI-related academic misconduct relied on self-reported data, which may not fully capture the complexity of how generative AI is used in real academic practice. Future research could address this limitation by employing scenario-based instruments, policy-specific vignettes, or behavioral assessments to more precisely differentiate clearly prohibited misconduct from ethically ambiguous GAI-supported academic practices.

Second, the GAI academic misconduct scale employed in this study, although adapted from a previously validated instrument, may not fully capture more nuanced or rapidly evolving forms of GAI misuse. As generative AI technologies continue to develop, students’ ways of engaging with these tools—such as iterative prompting, collaborative human-AI writing, or partial AI-assisted idea generation—are becoming increasingly complex and difficult to classify within fixed questionnaire items. Consequently, the current scale may lag behind emerging practices or oversimplify the spectrum of GAI-supported academic behaviors. While the use of a validated instrument enhances comparability and reliability, future research would benefit from continuously updating measurement tools or integrating mixed-method approaches to better reflect the dynamic and evolving nature of GAI-related academic misconduct. In addition, as with many survey-based studies, the present research relies on self-reported data collected at a single time point, which may raise concerns about common method variance (CMV). However, formal CMV diagnostics are mainly designed for variable-centered analyses, whereas this study adopts a configurational fsQCA approach that focuses on combinations of conditions rather than linear relationships among variables. Consequently, CMV is less likely to systematically bias the identified solution paths. Future studies could further reduce potential method-related bias by using multi-source or longitudinal data.

A further limitation of this study is that differences across academic levels were not examined. Students at different stages of study may vary in their exposure to academic integrity training, familiarity with institutional regulations, and understanding of the consequences of academic misconduct, particularly in relation to emerging technologies such as generative AI. As a result, perceptions of what constitutes inappropriate or unauthorized GAI use may differ across student populations. Future research could address this limitation by conducting stratified analyses or comparative studies across academic levels. Finally, the study relies on cross-sectional self-report data from Chinese university students, which may limit causal inference and generalizability to other contexts. In addition, only individual personality traits were examined, and future research could incorporate longitudinal designs, cross-cultural samples, and contextual factors to further clarify GAI-related academic misconduct.

## Conclusion

This study advances current understanding of academic integrity in the era of generative artificial intelligence by revealing how distinct personality configurations shape ethical and unethical academic behaviors. Using fsQCA, we identified multiple, equifinal pathways that lead to either high or low levels of GAI-related misconduct. These results underscore that integrity in AI-mediated learning is not determined by any single trait but emerges from interacting patterns of moral, self-regulatory, and dark personality characteristics.

Theoretically, the study integrates perspectives from moral psychology, self-regulation, and Dark Triad research into a unified configurational framework, highlighting the multidimensional nature of ethical decision-making in digital contexts. Practically, the findings suggest that universities might valuably move beyond punitive or technical solutions and instead foster character-based and reflective education—strengthening students’ honesty, conscientiousness, and empathy while discouraging manipulative or callous orientations.

In an age where GAI is reshaping academic practice, understanding the psychological foundations of ethical engagement becomes critical. By identifying the constellations that promote or inhibit misconduct, this study provides actionable insight for designing interventions that cultivate responsible, integrity-driven use of AI in higher education.

## Supplementary Information

Supplementary Material 1.

## Data Availability

The anonymized data analyzed in the current study are available from the first author on reasonable request.
